# Commentary: Evolution and hotspots in breast cancer organoid research: insights from a bibliometric and visual knowledge mapping study (2005–2024)

**DOI:** 10.3389/fonc.2025.1728125

**Published:** 2026-02-06

**Authors:** Xianlin Luo, Zaixiang Zhang, Hua Zhao

**Affiliations:** Hubei University of Chinese Medicine Affiliated Gong’an Hospital of Traditional Chinese Medicine, Jingzhou, China

**Keywords:** 3Dbioprinting, bibliometrics, breast cancer, drug discovery, organoids, research hotspots, tumor microenvironment

## 1 Introduction

In recent years, the exponential growth of biomedical literature has led to increasing recognition of bibliometrics as a robust method for quantitatively and qualitatively assessing research trends and emerging hotspots within specific scientific domains. We read with great interest the article by Tao Wu et al. (1), titled “Evolution and hotspots in breast cancer organoid research: insights from a bibliometric and visual knowledge mapping study (2005–2024),” published in Frontiers in Oncology. We commend the authors for their rigorous work and acknowledge their valuable contributions to advancing scholarship in this field.

## 2 Commentary and discussion

By using three bibliometric tools (VOSviewer, R-bibliometrix, and CiteSpace), this study conducted an in-depth analysis of the dynamic evolution of breast cancer organoid research over the past two decades. The finding provided a thorough summary of the major achievements, persistent challenges, and future frontiers within this rapidly advancing field. Key achievements encompass the successful implementation of patient-derived organoids (PDOs) for personalized drug testing and disease modeling, significant progress in recapitulating the tumor microenvironment and immune interactions, and the integration of innovative 3D bioprinting and engineering approaches. However, we identified several points requiring clarification and correction.

**Figure 3:**
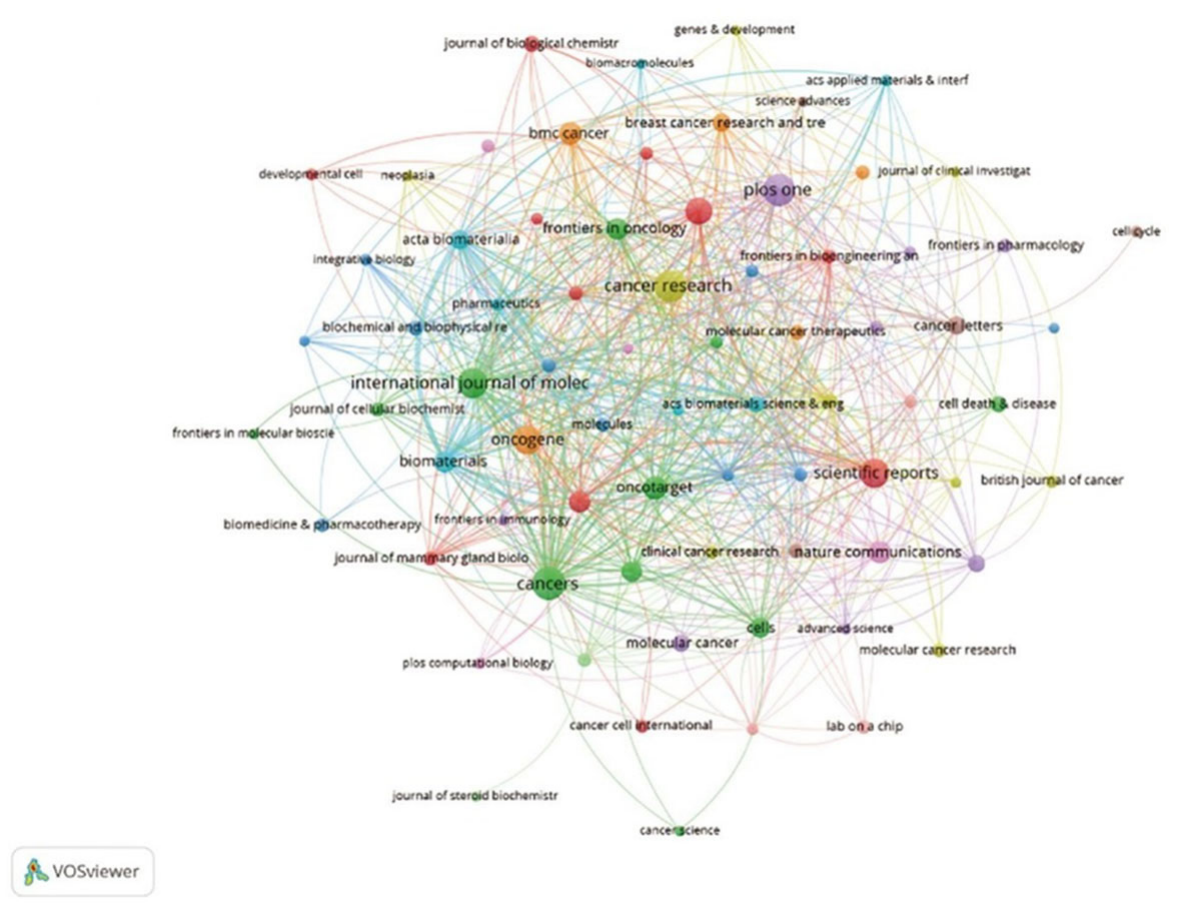
Institutional cooperation diagram based on VOSviewer.

First, regarding the Countries/regions and institutions analysis section: The text states: “the United States (666), China (257), India (106), the United Kingdom (105), and Germany (101) contributed the most.” However, the data presented in **Table 1** clearly indicate that Italy has 106 publications, not India. Consequently, the subsequent statement, “the United Kingdom, Germany, and the United States have higher citation rates than China and India,” should also be corrected by replacing “India” with “Italy.” Additionally, the text interprets **Figure 3A** as “a VOSviewer chart of institutional collaboration, American institutions, centered on Harvard University, work more closely together…” A review of **Figure 3A** indicates it is unambiguously a journal co-occurrence network map, not an institutional collaboration map. Therefore, the analysis pertaining to institutional collaboration is misplaced, and **Figure 3A** should be replaced with the correct institutional collaboration network diagram to support the textual argument.

**Table 1:** Top 10 producing countries related to breast cancer organoids.

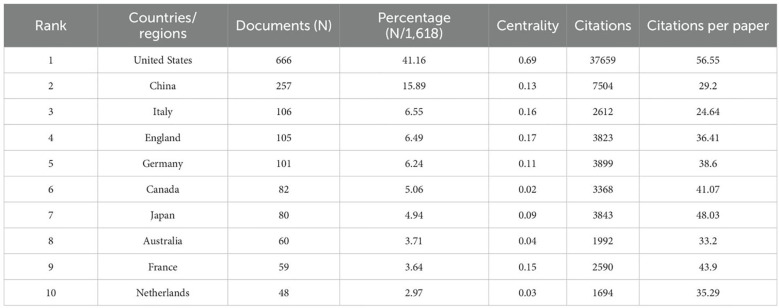

Second, regarding the Journal analysis section: The text claims: “8 journals had impact factors (IF) of more than 1.5.” This is inconsistent with the data provided in **Table 4** (“The top 10 journals in terms of publication volume, correlation strength, and citation times”), which shows that all 10 listed journals have impact factors exceeding 1.5, and 8 of them have impact factors greater than 3. The stated sentence requires correction to accurately reflect the data in the corresponding table. Authors can choose between two statements: “8 journals have an impact factor exceeding 3” or “all 10 listed journals have an impact factor exceeding 1.5.”

**Table 4:** Top 10 producing countries related to breast cancer organoids.

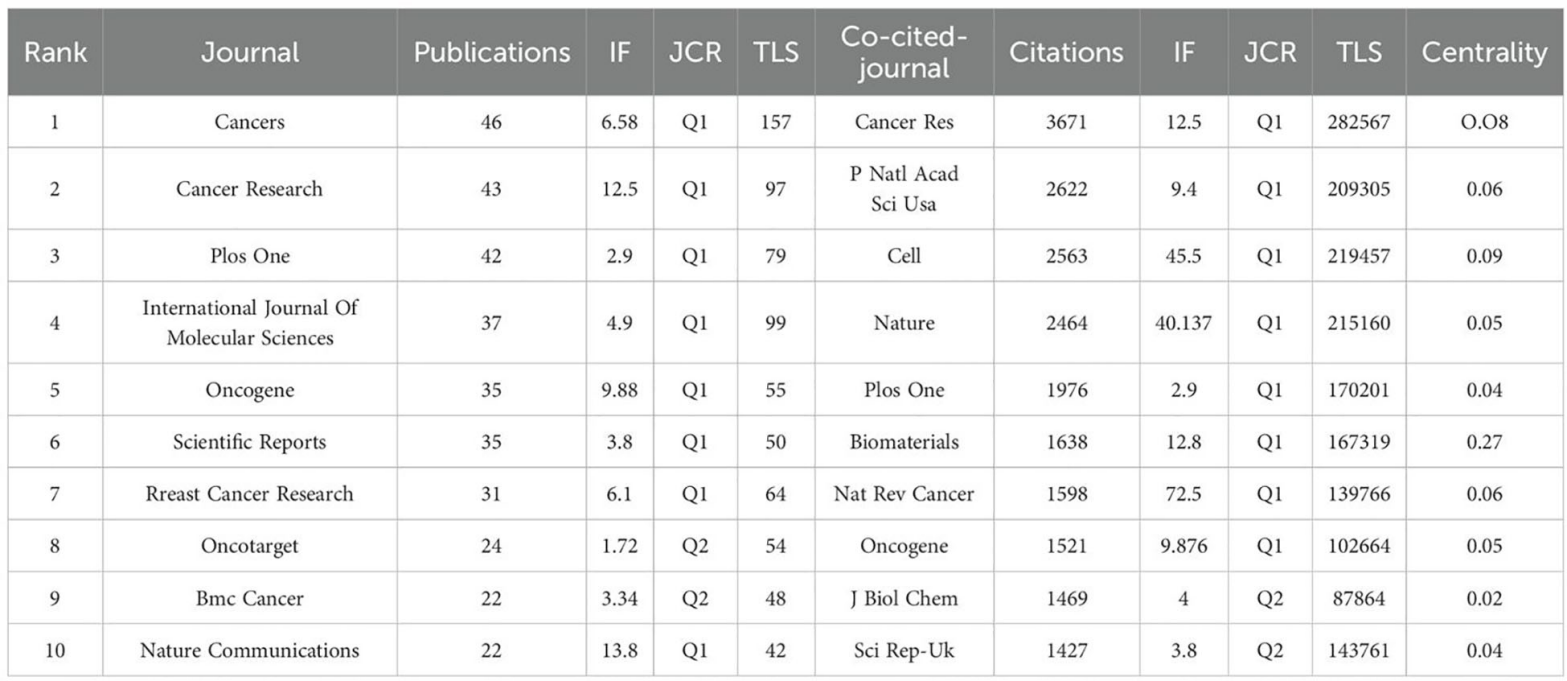

**Figure 6:**
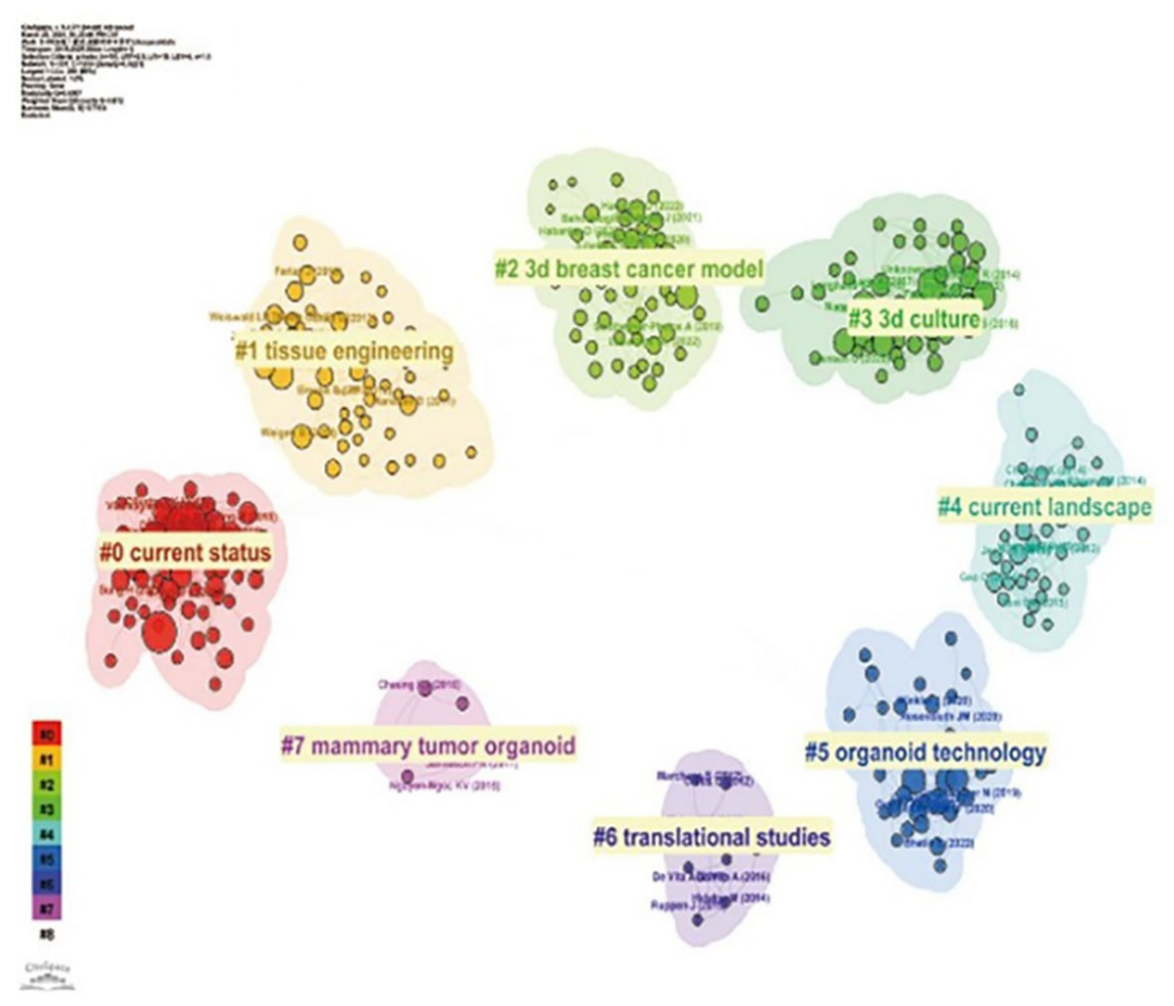
Cluster view of breast cancer organoids co-cited literature.

Third, regarding the Keyword analysis section: The text describes Figure 8B as a keyword cluster map generated by CiteSpace: “we use CiteSpace to group keywords into different clusters… (Figure 8B).” However, Figure 82B appears to be identical to **Figure 6B** (“Cluster view of breast cancer organoids co-cited literature”). As this figure does not represent a keyword cluster analysis, it is unsuitable for this section. Figure 8B should be replaced with the correct keyword clustering network map to substantiate the analysis of thematic evolution and research directions.
